# Personality traits and physical functioning: a cross-sectional multimethod facet-level analysis

**DOI:** 10.1186/s11556-020-00251-9

**Published:** 2020-11-24

**Authors:** Tiia Kekäläinen, Antonio Terracciano, Sarianna Sipilä, Katja Kokko

**Affiliations:** 1 Gerontology Research Center and Faculty of Sport and Health Sciences, University of Jyväskylä, Jyväskylä, Finland; 2 Department of Geriatrics, College of Medicine, Florida State University, Tallahassee, FL USA

**Keywords:** Physical activity, Accelerometer, Walking speed, Mobility

## Abstract

**Background:**

This study aimed to investigate whether personality traits and their facets are associated with a multi-methods assessment of physical activity and walking performance and whether they explain the discrepancy between self-reported and accelerometer-assessed physical activity.

**Methods:**

The participants were community-dwelling, 70–85-year-old men and women from Finland (*n* = 239) who were part of a clinical trial. Personality traits and their facets were measured using the 240-item NEO Personality Inventory-3. Physical activity was assessed using questions about frequency, intensity and duration of exercise (self-reported metabolic equivalent minutes (MET)) and by tri-axial accelerometers (light and moderate-to-vigorous physical activity and total MET-minutes). Walking performance was measured by 6-min walking distance and 10-m walking speed. Linear regression analyses were controlled for age, sex, education, body mass index, disease burden, and intervention group.

**Results:**

The *activity* facet of extraversion was positively associated with self-reported MET-minutes, accelerometer-assessed light physical activity and walking performance. The *positive emotions* facet of extraversion was positively associated with self-reported MET-minutes and walking performance. Openness and its facets and the *excitement seeking* facet of extraversion were positively associated with walking performance. Conscientiousness and most of its facets were associated with both physical activity and walking performance, but these associations were not statistically significant after accounting for all control variables. The *impulsiveness* facet of neuroticism was negatively associated with accelerometer-assessed light physical activity and walking performance, but the associations with walking performance attenuated after accounting for all control variables. Accelerometer-assessed moderate-to-vigorous physical activity was not associated with personality traits or facets. Discrepancy analyses suggest that openness and the *excitement-seeking* facet of extraversion were associated with higher self-reported than accelerometer-assessed physical activity.

**Conclusions:**

Consistently across methods, older adults who scored higher on facets of extraversion and conscientiousness tended to be more active and outperformed peers on walking performance. Older adults who scored higher in the facets of openness and the *excitement-seeking* facet of extraversion had better walking performance but also overestimated their self-reported physical activity compared to the accelerometers.

**Supplementary Information:**

The online version contains supplementary material available at 10.1186/s11556-020-00251-9.

## Background

Physical activity has many health benefits throughout the lifespan, but especially in old age it provides protection against common diseases and frailty [1]. Nevertheless, only a minority of older adults meet the recommended level of physical activity and activity tends to decrease with age [2]. Walking is the most common physical activity among older adults [3] and decline in walking speed predicts a decline in physical activity levels [4]. The large variability in physical activity levels is in part a reflection of individual differences in personality, defined as the typical pattern of feeling, thinking and behaving that characterize a person [5]. Using the Five Factor Model of personality (also known as Big Five; Table 1), several studies have found that people who score higher in extraversion, conscientiousness and openness as well as lower in neuroticism report more physical activity [7–9], have a higher walking speed [10–13], and greater aerobic capacity [13]. The information about personality correlates of physical activity and walking performance helps to identify potential risk groups who need additional attention in promotion of physical functioning. Individual differences in personality traits are known to be relatively stable throughout the adult lifespan [14, 15] and in general, associations between personality traits and physical activity seem to be robust between different age groups [8, 16, 17]. While current evidence suggests robust associations between personality traits and self-reported physical activity and walking speed, few studies have assessed personality traits at the level of facets (i.e., narrowly defined traits that compose each of the five broad traits; Table 1), and even fewer studies in the personality research have assessed physical activity using accelerometers. To our knowledge, no study has integrated this fragmented literature by examining the associations between personality facets and multiple measures of physical activity and walking performance in one sample.

**Table 1:** The Five Factor Model personality traits and their facets according to NEO-PI-3 [6]

Neuroticism	Extraversion	Openness	Agreeableness	Conscientiousness
N1 Anxiety	E1 Warmth	O1 Fantasy	A1 Trust	C1 Competence
N2 Angry Hostility	E2 Gregariousness	O2 Aesthetics	A2 Straightforwardness	C2 Order
N3 Depression	E3 Assertiveness	O3 Feelings	A3 Altruism	C3 Dutifulness
N4 Self-Consciousness	E4 Activity	O4 Actions	A4 Compliance	C4 Achievement-Striving
N5 Impulsiveness	E5 Excitement Seeking	O5 Ideas	A5 Modesty	C5 Self-Discipline
N6 Vulnerability	E6 Positive Emotions	O6 Values	A6 Tender-Mindedness	C6 Deliberation

As Table 1 illustrate, each personality factor is composed by related but distinct facets [6], and analyses at the more granular facet level can provide a more precise and deeper understanding of the associations between personality and outcomes [18–21]. An investigation at the facet-level can identify which specific facet is responsible for an association observed with the broader trait. It is possible that all facets of a trait have similar associations with an outcome, or the associations are driven by just one or a few facets, or there could be contrasting effects among facets of the same trait that could be masked by null associations at the trait level. Facet-level research can potentially improve prediction models and inform on the specific mechanisms linking psychological traits to physical activity. Research to date at the facet level suggests that the most robust positive associations are found between the *activity* facet of extraversion and physical activity [16, 22–28], which may even entirely explain the relationship between extraversion and physical activity [25]. The *activity* facet is also associated with walking speed [13]. This is not surprising because people who score high in *activity* tend to be busy, have a lot of energy and live at a rapid tempo [6]. The evidence for the other facets is more mixed, but, for example, the *self-consciousness* facet of neuroticism has been associated with lower levels of physical activity [23, 28, 29], the *self-discipline* facet of conscientiousness with higher physical activity [23, 24, 29] and the *dutifulness* facet of conscientiousness with higher physical activity [23] and better walking performance [13]. Most of the previous studies with self-reported physical activity have analyzed only some of the facets [24, 28, 29] and focused on younger adults [16, 24–27].

Most of the research about personality and physical activity is based on self-reported physical activity and the use of multiple measures of physical activity is necessary especially when studying older adults. Among older adults, most of the daily activity comes from light intensity activities that are easily under-reported in questionnaires [30] and, therefore, use of accelerometers can offer new information about associations between personality traits and physical activity among older adults. Accelerometers record body acceleration which is further transformed into other units, such as average daily time spend in different intensities of physical activity [31]. They can offer detailed information about daily physical activity and also overcome some common disadvantages of self-reports, such as recall bias [31]. Three previous studies on personality traits and accelerometer-assessed physical activity among older adults found mixed results: Artese and colleagues [23] found that extraversion, conscientiousness and agreeableness were positively and neuroticism negatively associated with physical activity outcomes, whereas Čukić and colleagues [32] with five personality traits and our own study with extraversion and neuroticism [33] found no associations between personality traits and accelerometer-based physical activity outcomes. However, among older adults, accelerometers may underestimate the amount of moderate-to-vigorous physical activity because of lower walking speed [34]. Therefore, it would be important to study walking performance together with different measures of physical activity.

The correlation between self-reports and accelerometers vary between studies but is usually low or at most moderate [35]. Even though the discrepancy between self-reported and accelerometer-assessed physical activity is well-known, individual characteristics that might explain this discrepancy are less studied. Current evidence suggests that age, sex, education and BMI are related to the discrepancy [36–38]. To our knowledge, our own previous study is thus far the only one to investigate personality and the discrepancies between physical activity measurements. Our results showed that older adults higher in neuroticism were more likely to underreport their physical activity compared to the accelerometer data [33].

The purpose of this study was to investigate whether personality traits and their facets 1) are associated with accelerometer-assessed and self-reported physical activity and walking performance, and 2) explain the discrepancy between accelerometer-assessed and self-reported physical activity. We expected that extraversion, openness and conscientiousness have positive associations and neuroticism a negative association with physical functioning. In the facet level, the strongest associations was expected to found between the *activity* facet of extraversion and physical functioning. For other facets the approach was exploratory, because to our knowledge, there is only one previous study about associations of all facet-level personality traits with walking performance [13] and one with accelerometer-assessed physical activity [23]. By addressing these questions, we aim to integrate evidence from multiple methods to advance knowledge on the role of personality traits in older adults’ physical functioning.

## Methods

### Participants

This study examined cross-sectional data collected in a post-intervention assessment of a randomized controlled trial “Promoting safe walking among older people: the effects of a physical and cognitive training intervention vs. physical training alone on mobility and falls among older community-dwelling men and women” (the PASSWORD study) [39]. The study was approved by the Ethics committee of the Central Finland Health Care District and all participants provided their written informed consent for participation. The study has been registered in the International Standard Randomized Controlled Trial Number Register: http/www.isrctn.com/ISRCTN52388040.

The participants were randomly selected from the Finnish National Registry. Recruitment started with an information letter sent to a random sample of 70- to 85-year-old community-dwelling older adults living in the city of Jyväskylä, Finland, followed up by a phone call. During the phone interview, willingness to participate in the study was confirmed and participants were screened for inclusion and exclusion criteria. The inclusion criteria at baseline were being at most moderately active (walking < 150 min/week, no regular resistance training), able to walk 500 m without assistance and to score ≥ 24 points on the Mini Mental State Examination. Exclusion criteria included severe chronic condition, medication, or other factors that may affect study participation, excessive alcohol use, difficulties in communication due to severe hearing or vision problems and another family member participating in the study [39]. The sample size was based on a pre-trial power analysis for primary outcomes of the trial (walking speed and falls rate) [39]. Participants were randomized to two groups, a combined physical and cognitive training vs physical training alone. As such, both groups participated in the same 12-month physical training intervention and both groups engaged in levels of physical training that matched the recommended guidelines including aerobic training (mostly walking), progressive resistance and balance training [39].

We have previously reported the baseline associations of extraversion and neuroticism (measured using a modified version of the Eysenck’s short Personality Inventory) with physical activity in the same sample [33]. This study used data only from post-intervention measurements because the NEO-Personality Inventory-3 (NEO-PI-3) was administered only in the post-intervention. The analyses were restricted to those participants (*n* = 239) who completed the NEO-PI-3. All of these participants had participated in physical training alone or combined physical and cognitive training during the last 12 months. Compared to the rest of the original sample (*n* = 75), the participants included in this study (*n* = 239) were younger (mean age 74.20 ± 3.71 vs. 75.33 ± 4.05, *p* = .021) and had a higher level of education (88% vs. 73% in the medium or high education group, *p* = .007).

### Measurements

The NEO-PI-3 was used to assess personality traits and facets [6, 40]. It has 240 items, 48 for each personality trait and 8 for each facet (see Table 1). The response scale is from 0 = strongly disagree to 4 = strongly agree. Participants were given a paper version of the NEO-PI-3 in the last training session and asked to return it when they came for the post-intervention measurements. Of 289 participants, 244 participants returned the NEO-PI-3 questionnaire. Three questionnaires were empty, and two participants had more than 40 missing values (the exclusion limit in the NEO-PI-3 manual [40]), so those participants (*n* = 5) were excluded from this study. Of those 239 participants who had filled the NEO-PI-3 questionnaire at least partly, 193 (81%) had filled it completely without any missing values and the rest of the participants (*n* = 46) had seven missing values at the most. These were imputed with the neutral option 2 [40]. Sum scores for each facet and each trait were calculated and these raw scores were used in the analyses. Raw scores were transformed to T-scores using combined sex-norms reported in the NEO-PI-3 Finnish version manual [40] for comparison purposes. The Cronbach’s alphas were 0.87 for neuroticism, 0.80 for extraversion, 0.75 for openness, 0.76 for agreeableness and 0.85 for conscientiousness.

Physical activity was assessed by both accelerometers and self-reports. Participants were asked to wear a tri-axial accelerometer, model UKK RM42 (UKK, Tampere, Finland), in an elastic band on their right hip during waking hours for seven consecutive days except during water-based activities [41]. The accelerometer stores acceleration at 100 Hz sampling rate with 13-bit A/D conversion of the ±16 g range. The raw acceleration data were analyzed with a custom-written MATLAB (version R2016b, The MathWorks Inc., Natick MA, USA) script for mean amplitude deviation (MAD) with a previously published algorithm [42]. Mean daily minutes for sedentary, light, moderate and vigorous intensity activity were calculated with previously validated cut-off points [42, 43]. Acceptable accelerometer data (at least 3 days with at least a 10-h wearing time) were available for 266 participants (wearing time mean 13.9 h/d ± 1.3, 90% of participants with 6 or 7 valid days). In the present study, information about light, moderate and vigorous activity was used. Because the amount of vigorous intensity activity was almost non-existent (mean 0.11 min/d ± 0.57), it was merged with moderate activity (MVPA). Three questions asking monthly frequency, duration and intensity of exercise were used to calculate daily self-reported MET-minutes [44].

For discrepancy analysis, average daily metabolic equivalent (MET)-minutes from accelerometer data were calculated with the common thresholds for light, moderate and vigorous activity: 1.6* light + 3*moderate + 6*vigorous physical activity [45]. Because MET-minutes from accelerometer (all daily activity) and self-reports (questions about exercise) were not directly comparable, standardized values were used. Standardized MET-minutes from accelerometer data were subtracted from standardized self-reported MET-minutes and such difference was used as the outcome in the analyses. The positive discrepancy score indicates that a participant had reported higher MET-minutes than what was assessed by the accelerometers compared to the other participants.

Two walking performance tests were used in this study: the 6-min walking test evaluates community walking [46] and the 10-m walking test evaluates maximum walking speed. In the 6-min walking test, participants walked up and down a 20-m circuit without resting. They were encouraged to walk as far as possible for 6 min [46]. In the 10-m walking test, participants were asked to walk as fast as possible over the 10-m course [39]. The time was measured by photocells and the maximum walking speed (m/s) was calculated from the best performance of two attempts.

The background variables included sex, age, body mass index (BMI), education and chronic diseases. Sex and date of birth were drawn from the Finnish Population Registry. BMI was calculated from staff-measured height and weight. Education was categorized to low (at most primary school), medium (middle school, folk high school, vocational school or secondary school) and high (college or university degree). Self-report chronic diseases diagnosed by physicians were reported at baseline and were updated with new verified diagnoses that occurred during the intervention. Answers to questions on chronic diseases were categorized into metabolic, cardiovascular, pulmonary, musculoskeletal, neurological and mental diseases. Number of disease categories was calculated and used in analyses with a categorization of 0, 1, 2 and 3 or more disease categories.

### Statistical analyses

All statistical analyses were carried out using SPSS, version 25 (IBM Corp., Armonk, NY). Demographic characteristics of the study sample were described using proportions, means and standard deviations and minimum and maximum values.

Associations of personality traits and their facets with physical activity and walking speed were analyzed by linear regression. Every trait and facet were analyzed in separate models to avoid multicollinearity. The models with all five traits in the same model are presented in supplementary material (see Additional file 1, Table S1). Sex, age, BMI, education and chronic diseases were used as covariates in line with previous studies assessing associations between personality traits and physical performance [8, 10, 13, 23, 32].

Linear regression models for each physical activity and walking performance outcomes as well as to the discrepancy score were computed in two phases: the first model (M1) included a trait or facet and sex and age as covariates while the rest of the covariates (BMI, chronic diseases, education and the intervention group) were included in the second model (M2). M2 models for light physical activity also included MVPA as a covariate and M2 models for MVPA also included light physical activity as a covariate. Because the variable had a right-skewed distribution in self-reported MET-minutes, the logarithm transformation was used in linear regression analysis.

The Benjamini-Hochberg procedure was used to correct the high number of comparisons with a false discovery rate of 0.10 [47, 48]. The procedure was conducted first to models with age and sex as covariates (M1) with six outcomes (walk 6 min, walk 10 m, light physical activity, MVPA, self-reported MET-minutes and discrepancy score) including a total of 210 models (6 outcomes* (5 traits + 30 facets)) and then similarly to 210 models with all covariates (M2).

## Results

The descriptive statistics for main study variables are presented in Table 2 and for the facets in supplementary material (Additional file 2, Table S2). A total of 141 (59%) women and 98 (41%) men with a mean age of 74.71 ± 3.72 and mean BMI of 27.76 ± 4.66 participated in this study. Most of the participants (66%) were in the medium education group, 12% had at most a primary degree (low) and 22% had a college or university degree (high). Only 13% did not report a chronic metabolic, cardiovascular, pulmonary, musculoskeletal, neurological or mental disease; 32% reported one, 32% reported two, and 23% reported three or more chronic disease categories. Compared to a Finnish normative sample [40], our sample of older adults tended to score low on neuroticism (T-score: 42.76 ± 7.84), high on agreeableness (58.16 ± 7.80), and about average on extraversion (49.50 ± 8.28), openness (48.58 ± 9.00), and conscientiousness (53.07 ± 9.29). The correlations between main study variables are presented in Table 3 and for facets in supplementary material (Additional file 3, Table S3).

**Table 2:** Descriptive statistics for study variables (*n* = 239)

	Mean ± SD	Min – Max
Accelerometer: Light physical activity min/d	206.56 ± 66.17	65.00–423.00
Accelerometer: MVPA min/d	32.15 ± 21.01	0–125.14
Accelerometer: MET min/d^a^	427.27 ± 129.84	107.00–786.89
Self-reported MET min/d	115.60 ± 108.09	0.47–750.00
6-min walking distance m	521.13 ± 92.76	249–736
10-m walking speed m/s	2.08 ± 0.42	0.96–3.22
Neuroticism	73.18 ± 21.82	17–133
Extraversion	95.11 ± 20.47	43–169
Openness	109.18 ± 18.98	63–181
Agreeableness	128.54 ± 15.94	77–170
Conscientiousness	116.42 ± 20.27	64–166

*MVPA* moderato-to-vigorous physical activity

^a^Calculated with a formula 1.6*light physical activity+ 3*moderate physical activity + 6*vigorous physical activity

**Table 3:** Pearson bi-variate correlations between study variables

	1.	2.	3.	4.	5.	6.	7.	8.	9.	10.	11.
**Accelerometer- based physical activity**											
1. Light											
2. MVPA	.12										
3. MET min	.87*	.59*									
**Self-reported physical activity**											
4. MET min	.25*	.40*	.40*								
**Walking tests**											
5. 6-min	.20*	.48*	.41*	.32*							
6. 10-m	.14*	.31*	.27*	.19*	.83*						
**Personality traits**											
7. Neuroticism	−.03	.02	−.02	−.12	−.21*	−.15*					
8. Extraversion	.16*	−.03	.11	.19*	.15*	.15*	−.30*				
9. Openness	.00	.05	.03	.13*	.14*	.12	.04	.42*			
10. Agreeableness	.01	−.05	−.01	.06	−.00	−.06	−.32*	−.01	.10		
11. Conscientiousness	.19*	.04	.18*	.21*	.24*	.17*	−.55*	.24*	−.11	.29*	
**Covariates**											
12. BMI	−.31*	−.14*	−.31*	−.18*	−.35*	−.19*	.04	.01	−.12	−.04	−.17*
13. Chronic diseases	.04	−.20*	−.07	−.08	−.27*	−.16*	.16*	.02	−.06	−.09	−.12
13. Age	−.08	−.28*	−.20*	−.22*	−.34*	−.30*	.12	−.10	−.00	.02	−.13*
14. Education	.11	.07	.12	−.00	.13*	.16*	.04	.08	.28*	−.08	−.06

*MVPA* moderate-to-vigorous physical activity, *MET* metabolic equivalent, *BMI* body mass index

*p < .05

### Physical activity

**Fig. 1:**
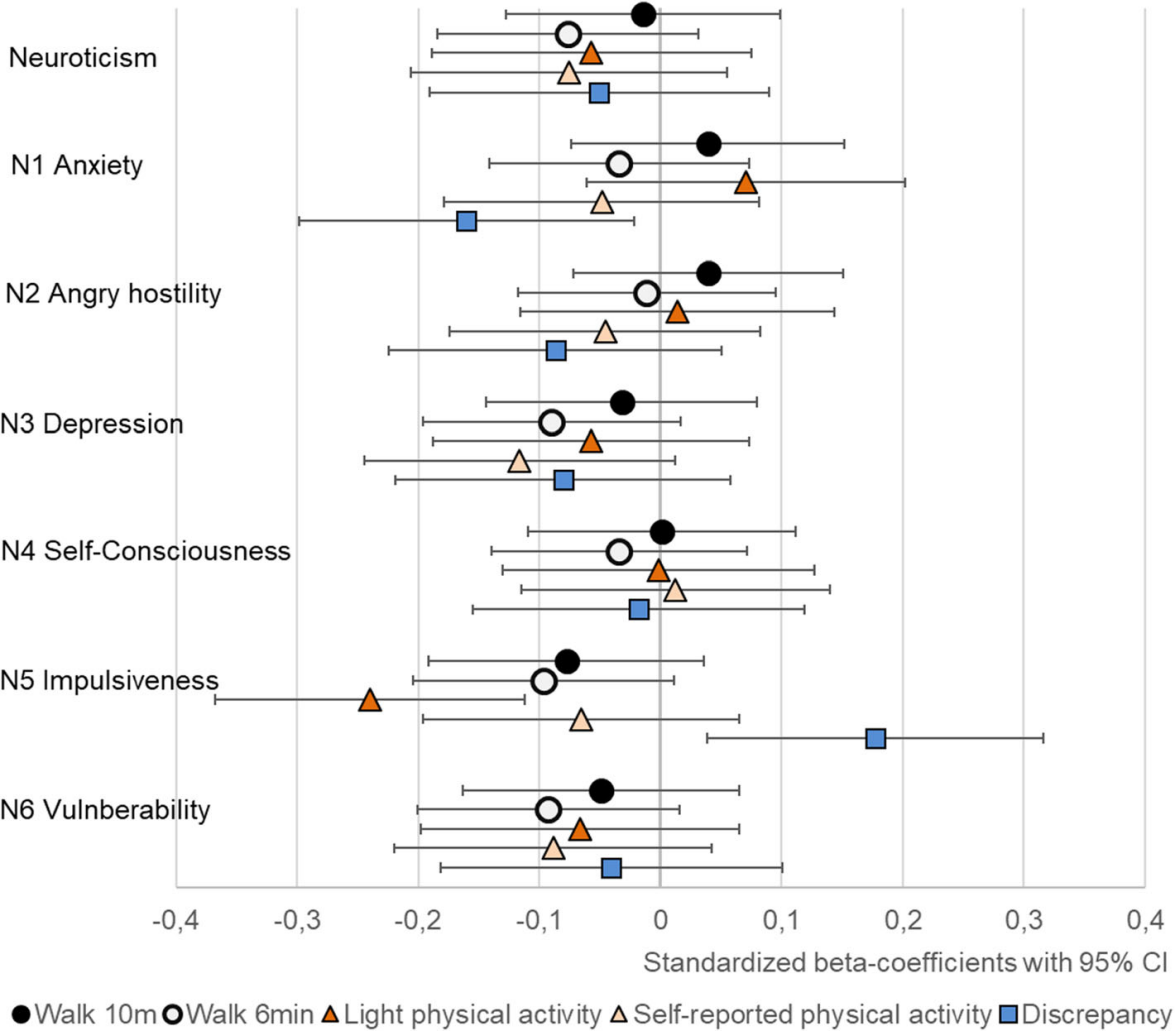
The associations of neuroticism with walking performance and physical activity (PA). Legend: Models adjusted by sex, age, education, BMI, diseases and intervention group, and for light PA also by moderate-to-vigorous physical activity

**Fig. 2:**
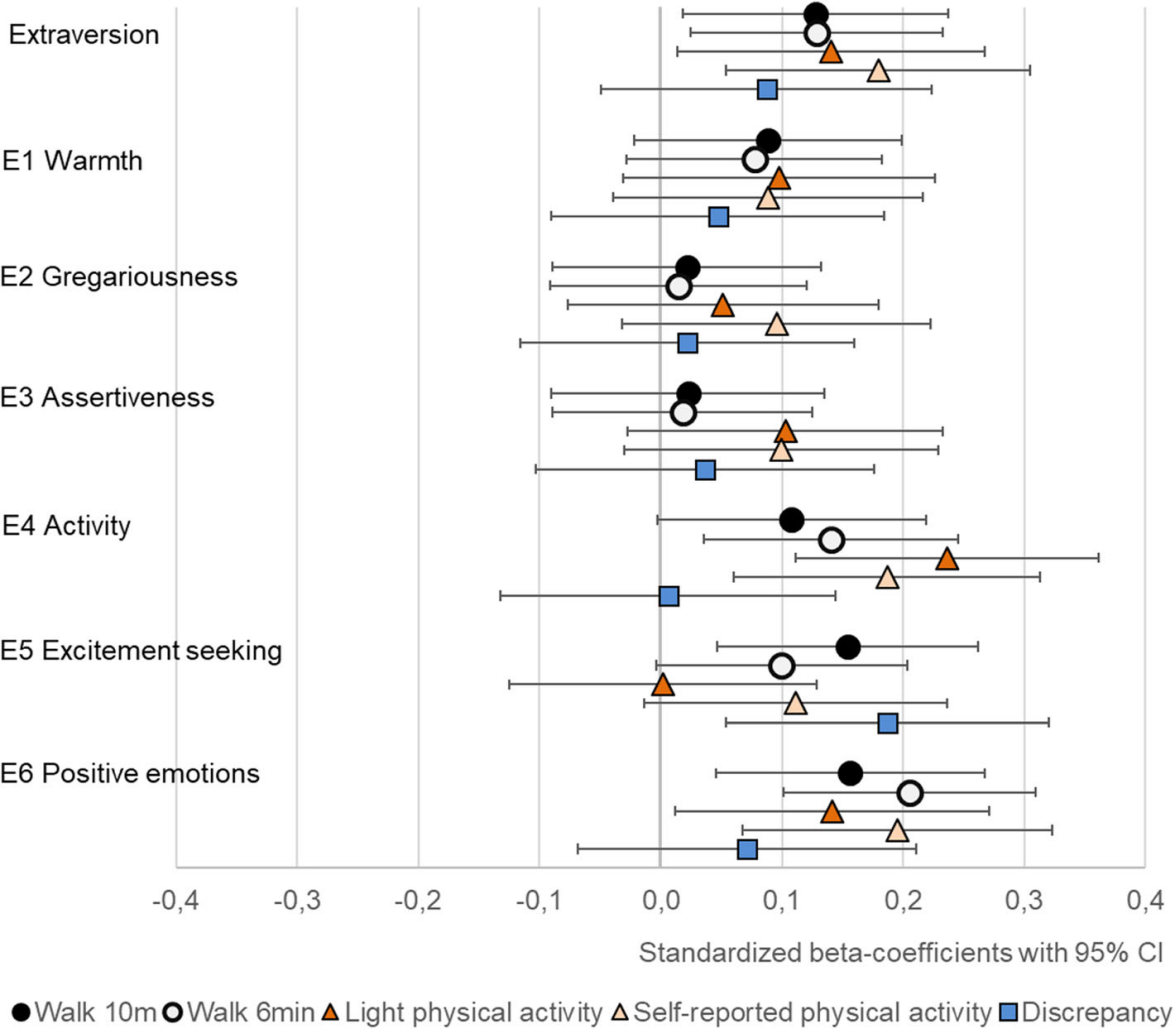
The associations of extraversion with walking performance and physical activity (PA). Legend: Models adjusted by sex, age, education, BMI, diseases and intervention group, and for light PA also by moderate-to-vigorous physical activity

**Fig. 3:**
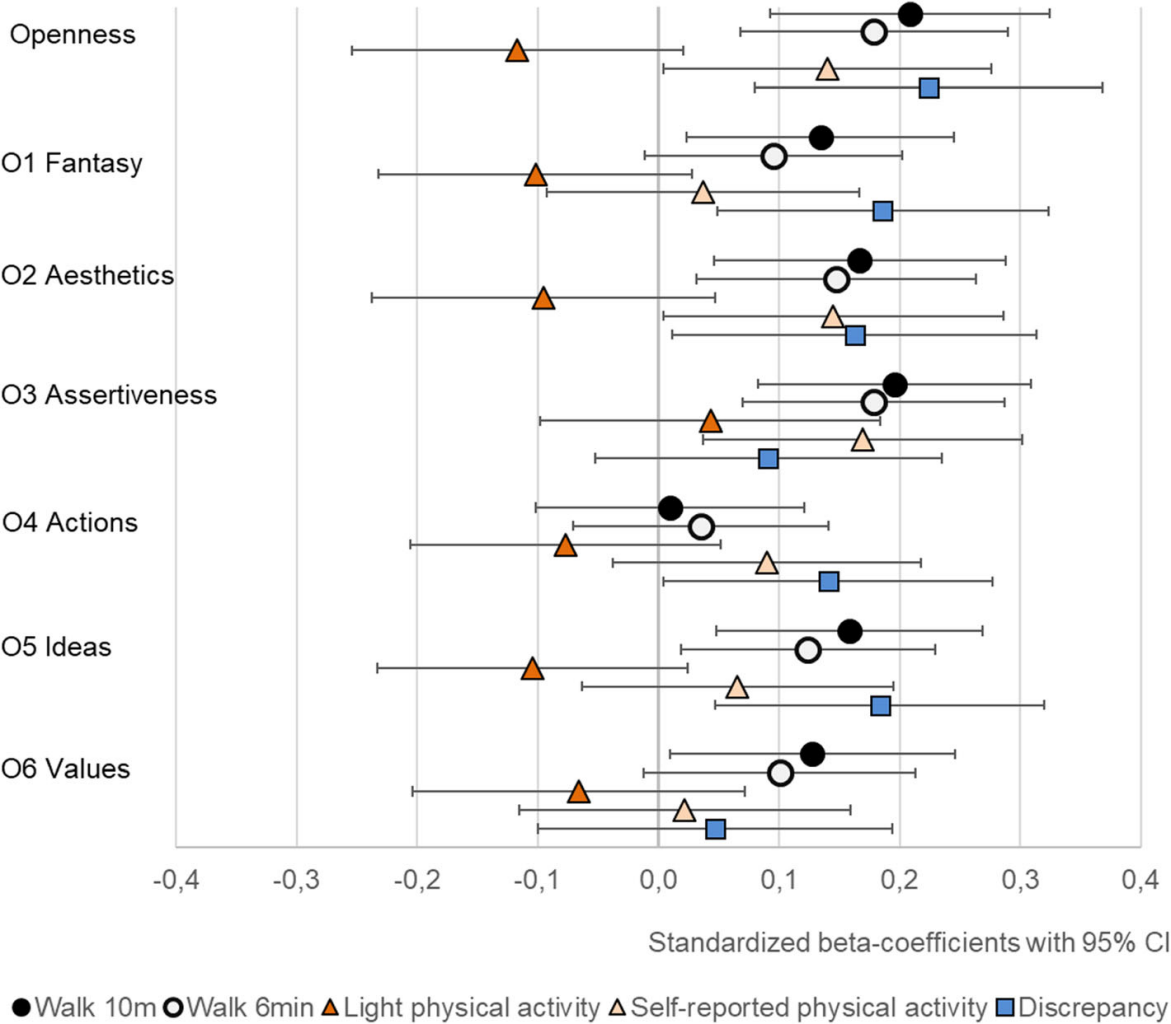
The associations of openness with walking performance and physical activity (PA). Legend: Models adjusted by sex, age, education, BMI, diseases and intervention group, and for light PA also by moderate-to-vigorous physical activity

**Fig. 4:**
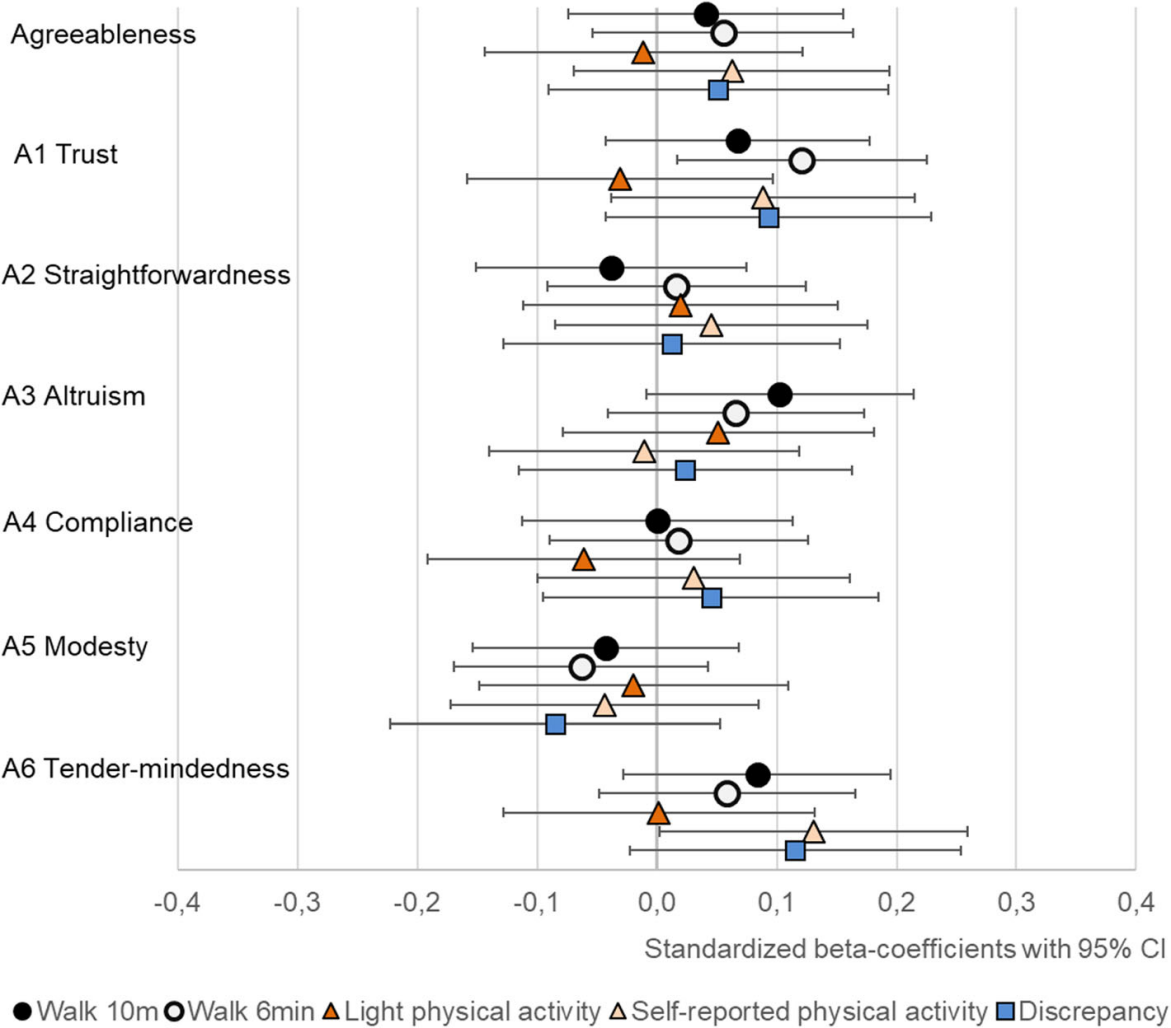
The associations of agreeableness with walking performance and physical activity (PA). Legend: Models adjusted by sex, age, education, BMI, diseases and intervention group, and for light PA also by moderate-to-vigorous physical activity

**Fig. 5:**
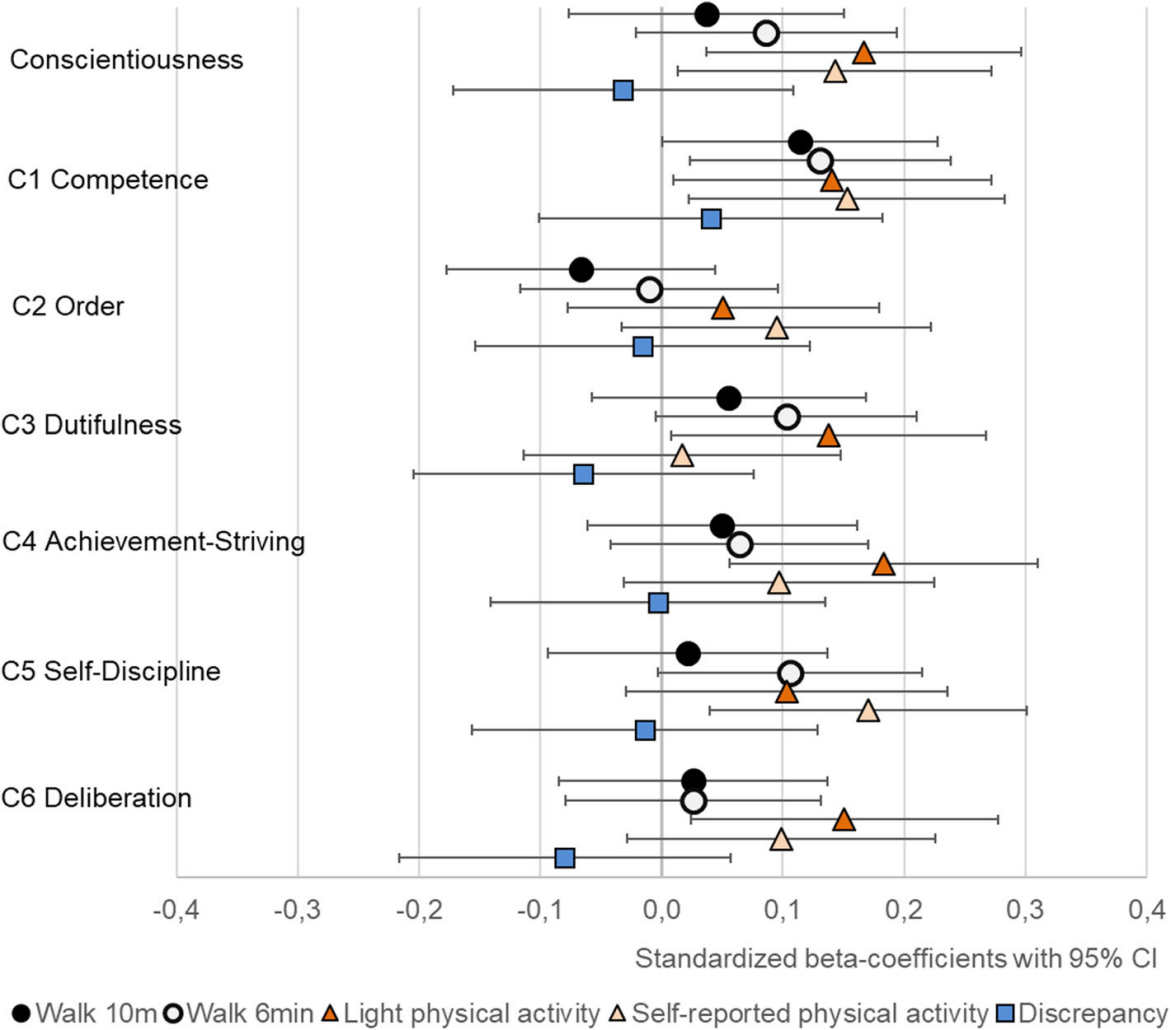
The associations of conscientiousness with walking performance and physical activity (PA). Legend: Models adjusted by sex, age, education, BMI, diseases and intervention group, and for light PA also by moderate-to-vigorous physical activity

At the broad five trait level, extraversion was positively associated with self-reported MET-minutes and conscientiousness with both light physical activity and self-reported MET-minutes (Table 4 and Figs. 1, 2, 3, 4 and 5). The association of conscientiousness with both outcomes became statistically non-significant in the model with all control variables (M2) after Benjamini-Hochberg corrections. Neuroticism and agreeableness were not associated with any physical activity outcomes. None of the traits was associated with MVPA (Additional file 4, Table S4).

**Table 4:** Associations of personality traits and facets with walking performance, physical activity and discrepancy between physical activity measurements

	Walking distance 6-min		Walking speed 10-m		Self-reported physical activity		Light physical activity		Discrepancy^a^	
	M1	M2	M1	M2	M1	M2	M1	M2	M1	M2
Neuroticism	−.13*	−.11	−.05	−.02	−.09	−.08	−.05	−.06	−.05	−.05
Extraversion	.14*	.13*	.15*	.13*	.17**	.18**	.15*	.14*	.08	.09
Openness	.25***	.18*	.27***	.21***	.15*	.14*	−.05	−.12	.19**	.22**
Agreeableness	.07	.06	.04	.04	.08	.06	−.02	−.01	.06	.05
Conscientiousness	.17**	.09	.09	.04	.18**	.14*	.20**	.17*	−.05	−.03
N1 Anxiety	−.03	−.03	.04	.04	−.04	−.05	.11	.08	−.18*	−.16*
N2 Angry Hostility	−.02	−.01	.04	.04	−.05	−.05	.05	.02	−.10	−.09
N3 Depression	−.15*	−.09	−.08	−.03	−.13*	−.12	−.05	−.05	−.08	−.08
N4 Self-Consciousness	−.08	−.03	−.04	.00	.01	.01	−.01	.01	−.01	−.02
N5 Impulsiveness	−.21***	−.10	−.15*	−.08	−.12	−.07	−.29***	−.24***	.19*	.18*
N6 Vulnerability	−.12	−.09	−.07	−.05	−.08	−.09	−.04	−.06	−.06	−.04
E1 Warmth	.06	.08	.08	.09	.08	.09	.06	.10	.06	.05
E2 Gregariousness	.00	.02	.03	.02	.08	.10	.03	.05	.03	.02
E3 Assertiveness	.05	.02	.08	.03	.09	.10	.12	.10	.02	.04
E4 Activity	.19**	.14*	.16**	.11	.20**	.19**	.28***	.24***	−.02	.01
E5 Excitement Seeking	.12*	.10	.18**	.15**	.12	.11	.03	.00	.17*	.19**
E6 Positive Emotions	.18**	.21***	.14*	.16**	.19**	.20**	.12	.14*	.07	.07
O1 Fantasy	.09	.10	.13*	.13*	.04	.04	−.09	−.10	.18**	.19**
O2 Aesthetics	.18**	.15*	.21**	.17**	.16*	.15*	−.02	−.09	.12	.16**
O3 Feelings	.19**	.18*	.21**	.19**	.16*	.17*	.04	.03	.08	.09
O4 Actions	.11	.04	.07	.01	.11	.09	−.03	−.08	.12	.14*
O5 Ideas	.19**	.12*	.23***	.16*	.07	.07	−.07	−.11	.16*	.18**
O6 Values	.20**	.10	.21***	.13*	.05	.02	.01	−.06	.02	.05
A1 Trust	.17**	.12*	.10	.07	.11	.09	−.01	−.03	.09	.09
A2 Straightforwardness	.08	.02	−.01	−.04	.08	.05	.05	.02	.00	.01
A3 Altruism	.02	.07	.07	.10	−.03	−.01	.02	.05	.04	.02
A4 Compliance	−.01	.02	−.04	.00	.03	.03	−.10	−.06	.07	.05
A5 Modesty	−.08	−.06	−.07	−.04	−.04	−.04	−.04	−.02	−.07	−.09
A6 Tender-mindedness	.08	.06	.11	.08	.14*	.13*	.02	.01	.11	.12
C1 Competence	.20**	.13*	.17**	.11*	.18**	.15*	.17*	.14*	.03	.04
C2 Order	.05	−.01	−.04	−.07	.13*	.10	.09	.05	−.04	−.02
C3 Dutifulness	.17**	.10	.10	.05	.04	.02	.15*	.14*	−.07	−.06
C4 Achievement-Striving	.13*	.06	.09	.05	.13*	.10	.23**	.18**	−.03	−.00
C5 Self-Discipline	.19**	.11	.07	.02	.21**	.17*	.14*	.10	−.03	−.01
C6 Deliberation	.10	.03	.07	.03	.13*	.10	.17**	.15*	−.09	−.08

Every trait and facet analyzed in the separate linear regression model; standardized beta-coefficients represented

^a^Standardized self-reported MET-minutes – standardized accelerometer-assessed MET-minutes. M1 = model including sex and age as covariates. M2 = model including sex, age, education, BMI, diseases and intervention group. M2 for light physical activity controlled also with moderate-to-vigorous physical activity

Bolded beta-coefficients remained statistically significant after Benjamini-Hochberg procedure.

***p < .001, **p < .01, *p < .05

At the facet level, higher scores in most facets of conscientiousness were associated with higher physical activity (Table 4, Fig. 5): C1 *competence* was associated with both self-reported MET-minutes and light physical activity, C4 *achievement-striving* and C6 *deliberation* with light physical activity and C5 *self-discipline* with self-reported MET-minutes. The inclusion of all control variables in M2 models reduced the associations of conscientiousness facets with both self-reported MET-minutes and light physical activity. After the Benjamini-Hochberg corrections, only C4 *achievement-striving* remained statistically significant predictor of light physical activity and none of the facets for self-reported MET-minutes. The E4 *activity* facet of extraversion was consistently associated with both light physical activity and self-reported MET-minutes and E6 *positive emotions* facet with self-reported MET-minutes in all models (Table 4, Fig. 2). Of other facets, N5 *impulsiveness* was negatively associated with light physical activity in both models. The only other facet-level association was found between O3 *feelings* and self-reported MET-minutes, but the associations became non-significant after accounting for all control variables (M2) and Benjamini-Hochberg corrections.

### Walking performance

Openness and most of its facets had the strongest associations with walking performance (Table 4 and Fig. 3). The extraversion facets E4 *activity*, E5 *excitement seeking* and E6 *positive emotions* had positive associations with walking performance tests, but E5 *excitement seeking* was associated only with walking speed and the association of E4 *activity* with walking speed was not statistically significant in M2 (Table 4, Fig. 2). The positive association between conscientiousness and its facets with walking distance did not remain statistically significant in M2. Similarly, the negative association between N5 *impulsiveness* and both walking performance tests became statistically non-significant after accounting for BMI, disease burden, education and intervention group. Of other facets, A1 *trust,* had positive association with walking performance in M1, but the associations became statistically non-significant in M2 after the Benjamini-Hochberg procedure (Table 4).

### Discrepancy

Participants who scored higher in openness (at least in O1 *Fantasy* and O5 *Ideas*) reported more physical activity than what was assessed by the accelerometers (Table 4, Fig. 3). None of the other traits was associated with discrepancy in the trait-level, but higher scores in N5 *impulsiveness* and E5 *excitement seeking* were associated with reporting more and N1 *anxiety* less physical activity compared to the accelerometers. The associations of N1 *anxiety* and N5 *impulsiveness* did not remain statistically significant in M2 after the Benjamini-Hochberg procedure.

## Discussion

Among the significant findings, the *activity* and *positive emotion* facets of extraversion and the facets of conscientiousness were positively associated with physical activity and walking performance. These findings suggest consistent associations with physical performance regardless of the way it is assessed. There were also contrasting findings across methods, and discrepancy analyses indicate that individuals who scored higher in openness and *excitement-seeking* tend to overestimate their physical activity levels using self-reports compared to the accelerometers but still had better walking performance. Next, we discuss the findings for each trait.

The negative association between neuroticism and physical activity found in previous studies [8, 9] was weaker and non-significant in the present study. The non-significant associations may be in part due to the lower scores in neuroticism of this sample compared to the Finnish adult population [40]. However, the facet-level analysis of neuroticism revealed some interesting findings. The *impulsiveness* facet had one of the strongest associations with physical activity: participants who scored higher in *impulsiveness* had lower accelerometer-assessed light physical activity and lower walking distance but did not have significantly lower self-reported physical activity. This discrepancy indicates that more impulsive individuals may over-report their level of physical activity. *Impulsiveness* is related to unfavorable health behaviors, such as obesity [49], and people who score high in *impulsiveness* might seek immediate rewards from eating or sedentary behavior rather than delayed ones from physical activity. Our previous results with the baseline sample using a modified version of the Eysenck’s short personality inventory showed that individuals who scored high in neuroticism were more likely to underreport their physical activity compared to the accelerometer data [33]. This result, which conflicts with the current findings for *impulsiveness* but accords with the findings for *anxiety*, highlights the importance of facet-level analysis.

Extraversion was consistently associated with physical activity and walking performance in line with previous studies [8–13, 23], but only the strongest association with self-reported physical activity remained statistically significant after the correction for multiple tests. In line with the expectations, the findings support the importance of the *activity* facet in the relationship between extraversion and physical activity [23–25, 28]. In addition to *activity*, *positive emotions* and *excitement-seeking* were linked to the outcomes: *positive emotions* with both self-reported physical activity and walking performance and *excitement-seeking* with walking performance and discrepancy. These three facets of extraversion describe the tendencies of being energetic and optimistic and seeking excitement and stimulus [6]. It seems that this side of extraversion is more important for physical performance than the tendencies related to social relationships captured by the three other facets of extraversion.

The results for openness in this study were in line with previous studies showing a weak positive association between openness and self-reported physical activity [8, 9] and no association with accelerometer-assessed physical activity [23]. Based on these results and the positive association between openness and discrepancy, individuals who scored higher in openness may overestimate their level of physical activity. However, older adults who scored higher in openness had better walking performance. Openness indicates willingness to try new things and individuals who score high in openness may engage in different types of physical activity [9]. The hip-worn accelerometer used in this study may not have captured all activities, such as strength training and cycling, and they were not worn during water-based activities. It is possible that this limitation of accelerometers explains the association between openness (and *excitement seeking*) and discrepancy.

Facets of conscientiousness were consistently related to physical activity and none of the facets stood out from the rest, supporting the previous studies about the positive association between conscientiousness and physical activity [7–9]. One explanation for this relationship is that individuals who are well-organized, dutiful, work hard to achieve their goals and have high self-discipline are more likely to implement their physical activity intentions [50]. Many facets of conscientiousness reflect self-control; hence, it is not surprising that conscientiousness has the opposite association with physical activity than impulsiveness. In contrast to impulsiveness, conscientiousness is linked to healthier weight [49, 51]. People with low conscientiousness participate less in physical activity, which may lead to higher BMI and, in turn, to poorer walking performance and health outcomes [52]. In line with some previous studies [9], agreeableness was not associated with physical activity or walking performance. There was also no robust evidence of associations at the facet level.

This study has some limitations. While this was one of the largest studies to report on the associations between personality traits and accelerometer-measures, the sample size provided limited power to detect the typical small effects. In our relatively small sample, only the largest effects remained significant after accounting for multiple-testing, increasing the risk of false negative. Hence, these exploratory findings should be replicated with other samples and also with corresponding samples that have not participated in a training intervention. In addition, owing to the cross-sectional nature of this study, we cannot draw any conclusions about the causal relationships between the study variables.

Accelerometers have some limitations related to the cut-off points for older adults and their capability to capture different types of physical activities [30, 31]. This might explain why we did not find an association with MVPA, which was surprising since the previous study on personality traits and accelerometer-assessed physical activity among older adults found associations only with MVPA and not with light physical activity [23]. Another explanation might be the low amount of MVPA among the participants of this study (mean 32.15 min/d ± 21.01). For example, older adults in the study by Artese et al. [23] had almost four times more MVPA per day on average (mean 113.3 ± 64.9). However, our sample of older adults did not differ so much from the Finnish adult population (aged 20–69) who has about 45 min of MVPA per day on average [53].

Accelerometers record body acceleration in all daily activities (when a participant wears the accelerometer) [31] whereas self-reported MET-minutes used in this study asked about participation in leisure time exercise [44]. Therefore, it is possible that the discrepancy in part reflects participating in exercise but having very little amount of other daily activity. In any case, the discrepancy variable in this study was based on the difference between standardized variables and therefore indicated whether a participant reported the same amount of physical activity that was captured by accelerometers compared to the other participants.

Despite these limitations, this study was one of the first to investigate the role of personality in the discrepancy between physical activity measures and contributes to our understanding of how personality is linked to different measures of physical activity and walking performance. The major strengths of this study were the use of multiple measures of physical activity and walking performance and an in-depth assessment of personality at both trait- and facet-levels. Another relatively unique feature of this study was the use of a post-intervention sample. Unfortunately, we did not have access to the NEO-PI-3 at baseline data collection and therefore data collection occurred one-year after a physical activity intervention was used. As such, the findings illustrate the role of individual differences in personality for physical functioning even after older adults engaged in a standardized intervention to increase mobility.

## Conclusions

This study has provided a deeper insight into how facet-level personality tendencies explain part of the variability in physical activity and walking performance. Older adults with higher impulsiveness and lower self-control, who are less energetic and have less optimistic tendencies are at a higher risk for physical inactivity and walking limitations. Testing for personality characteristics in health care settings could help to identify individuals with higher risk for limitations. Interventions to promote physical functioning could also benefit from a more individually tailored approach. However, more research is needed on effective ways to promote physical functioning among people with personality characteristics that increase the risk for low physical functioning. In addition, a greater focus on personality characteristics could produce interesting findings that help us understand the discrepancy between self-reports and activity monitors.

## Supplementary Information

**Additional file 1:** **Table S1.** Associations of personality traits and facets with physical activity, discrepancy between physical activity measurements and walking performance. Description: Results of the regression analyses were five personality traits were tested in the same regression models.

**Additional file 2:** **Table S2.** Descriptive statistics for facets. Description: Descriptive statistics for facets.

**Additional file 3:** **Table S3.** Pearson bi-variate correlations between personality facets and physical functioning. Description: Pearson bi-variate correlations between personality facets and physical functioning.

**Additional file 4:** **Table S4.** Associations of personality traits and facets with accelerometer-assessed moderate-to-vigorous physical activity. Description: Results of the regression analyses for accelerometer-assessed moderate-to-vigorous physical activity.

## Data Availability

Pseudonymized datasets are available on reasonable request. To request the data please contact Prof. Sarianna Sipilä (Sarianna.sipila@jyu.fi) (the PASSWORD data).

## Abbreviations

A1-A6: Facets of agreeableness
BMI: Body mass index
C1-C6: Facets of conscientiousness
E1-E6: Facets of extraversion
MET: Metabolic equivalent
MVPA: Moderate-to-vigorous physical activity
N1-N6: Facets of neuroticism
NEO-PI-3: NEO Personality Inventory − 3
O1-O6: Facets of openness
PASSWORD: Promoting safe walking among older people: the effects of a physical and cognitive training intervention vs. physical training alone on mobility and falls among older community-dwelling men and women
