# Supplementary material for: Personality traits and physical functioning: a cross-sectional multimethod facet-level analysis
Source: Eur Rev Aging Phys Act. 2020 Nov 24;17:20. doi: 10.1186/s11556-020-00251-9 (PMC7685629; doi:10.1186/s11556-020-00251-9)
Supplement: Supplementary file 1 — Additional file 1: Table S1. Associations of personality traits and facets with physical activity, discrepancy between physical activity measurements and walking performance. Description: Results of the regression analyses were five personality traits were tested in the same regression models. [file 11556_2020_251_MOESM1_ESM.docx]

Table S1. Associations of personality traits and facets with physical activity, discrepancy between physical activity measurements and walking performance.

|  | Walking distance  6-min | | Walking speed  10-m | | Light physical activity | | MVPA | | Self-reported  physical activity | | Discrepancy^a^ | |
| --- | --- | --- | --- | --- | --- | --- | --- | --- | --- | --- | --- | --- |
|  | M1 | M2 | M1 | M2 | M1 | M2 | M1 | M2 | M1 | M2 | M1 | M2 |
| Neuroticism | -.04 | -.01 | .03 | .05 | .11 | .07 | .09 | .11 | .05 | .05 | -.08 | -.06 |
| Extraversion | -.03 | .04 | .02 | .06 | .16* | .21** | -.11 | -.08 | .10 | .13 | .01 | -.01 |
| Openness | .28*** | .17* | .27*** | .19** | -.10 | -.20** | .16* | .12 | .13 | .09 | .17* | .22** |
| Agreeableness | -.02 | .02 | .01 | .04 | -.05 | -.01 | -.02 | .01 | .04 | .04 | .06 | .04 |
| Conscientiousness | .19** | .08 | .12 | .05 | .23** | .14 | .08 | .02 | .18** | .13 | -.10 | -.06 |

Traits analyzed in the same linear regression model; standardized beta-coefficients represented. MVPA = moderato-to-vigorous physical activity, ^a^= Standardized self-reported MET-minutes – standardized accelerometer-assessed MET-minutes. M1= model including sex and age as covariates. M2=model including sex, age, education, BMI, diseases and intervention group. M2 for light physical activity controlled also with MVPA and M3 for MVPA with light physical activity. *p<.05, **p<.01, ***p<.001.
