# Supplementary material for: Personality traits and physical functioning: a cross-sectional multimethod facet-level analysis
Source: Eur Rev Aging Phys Act. 2020 Nov 24;17:20. doi: 10.1186/s11556-020-00251-9 (PMC7685629; doi:10.1186/s11556-020-00251-9)
Supplement: Supplementary file 2 — Additional file 2: Table S2. Descriptive statistics for facets. Description: Descriptive statistics for facets. [file 11556_2020_251_MOESM2_ESM.docx]

Table S2. Descriptive statistics for facets

|  | Mean ± SD | Min - Max |
| --- | --- | --- |
| N1 Anxiety | 12.37 ± 5.40 | 0 – 28 |
| N2 Angry Hostility | 10.52 ± 4.39 | 1 – 25 |
| N3 Depression | 12.41 ± 4.76 | 2 – 31 |
| N4 Self-Consciousness | 12.23 ± 4.80 | 0 – 26 |
| N5 Impulsiveness | 14.65 ± 4.65 | 1 – 27 |
| N6 Vulnerability | 11.00 ± 4.20 | 0 – 24 |
| E1 Warmth | 21.74 ± 4.31 | 7 – 32 |
| E2 Gregariousness | 15.62 ± 5.10 | 2 – 32 |
| E3 Assertiveness | 13.99 ± 5.18 | 3 – 32 |
| E4 Activity | 15.05 ± 4.19 | 6 – 28 |
| E5 Excitement Seeking | 12.17 ± 4.74 | 0 – 25 |
| E6 Positive Emotions | 16.54 ± 5.36 | 0 – 30 |
| O1 Fantasy | 16.91 ± 5.13 | 2 – 32 |
| O2 Aesthetics | 20.20 ± 5.96 | 5 – 32 |
| O3 Feelings | 19.46 ± 4.49 | 8 – 32 |
| O4 Actions | 14.77 ± 4.18 | 2 – 26 |
| O5 Ideas | 18.13 ± 4.66 | 4 – 31 |
| O6 Values | 19.70 ± 3.77 | 12 – 31 |
| A1 Trust | 22.18 ± 3.88 | 9 – 30 |
| A2 Straightforwardness | 21.76 ± 4.57 | 9 – 32 |
| A3 Altruism | 22.20 ± 3.71 | 11 – 32 |
| A4 Compliance | 18.87 ± 4.19 | 7 – 29 |
| A5 Modesty | 20.56 ± 3.96 | 5 – 29 |
| A6 Tender-mindedness | 22.96 ± 3.72 | 13 – 32 |
| C1 Competence | 19.97 ± 3.96 | 8 – 32 |
| C2 Order | 19.39 ± 5.12 | 2 – 30 |
| C3 Dutifulness | 23.84 ± 3.70 | 11 – 32 |
| C4 Achievement-Striving | 15.38 ± 4.38 | 6 – 27 |
| C5 Self-Discipline | 19.56 ± 4.68 | 5 – 31 |
| C6 Deliberation | 18.28 ± 4.87 | 6 – 31 |
