# Supplementary material for: Personality traits and physical functioning: a cross-sectional multimethod facet-level analysis
Source: Eur Rev Aging Phys Act. 2020 Nov 24;17:20. doi: 10.1186/s11556-020-00251-9 (PMC7685629; doi:10.1186/s11556-020-00251-9)
Supplement: Supplementary file 3 — Additional file 3: Table S3. Pearson bi-variate correlations between personality facets and physical functioning. Description: Pearson bi-variate correlations between personality facets and physical functioning. [file 11556_2020_251_MOESM3_ESM.docx]

Table S3. Pearson bi-variate correlations between personality facets and physical functioning.

|  | Accelerometer | | | Self-reported | Walking tests | |
| --- | --- | --- | --- | --- | --- | --- |
|  | LPA | MVPA | MET min | MET min | 6-min | 10-m |
| N1 Anxiety | .13 | .06 | .13^*^ | -.06 | -.10 | -.06 |
| N2 Angry Hostility | .06 | .05 | .07 | -.07 | -.08 | -.03 |
| N3 Depression | -.05 | .03 | -.03 | -.16^*^ | -.22^*^ | -.16^*^ |
| N4 Self-Consciousness | -.01 | .05 | .02 | -.02 | -.13^*^ | -.10 |
| N5 Impulsiveness | -.28^*^ | -.07 | -.26^*^ | -.13^*^ | -.25^*^ | -.19^*^ |
| N6 Vulnerability | -.02 | -.04 | -.04 | -.11 | -.21^*^ | -.18^*^ |
| E1 Warmth | .08 | -.10 | .02 | .09 | .06 | .06 |
| E2 Gregariousness | .05 | -.06 | .01 | .09 | .01 | .02 |
| E3 Assertiveness | .12 | .01 | .11 | .11 | .10 | .13 |
| E4 Activity | .29^*^ | .05 | .26^*^ | .22^*^ | .22^*^ | .19^*^ |
| E5 Excitement Seeking | .01 | .04 | .03 | .11 | .14^*^ | .20^*^ |
| E6 Positive Emotions | .15^*^ | -.07 | .08 | .18^*^ | .13^*^ | .07 |
| O1 Fantasy | -.05 | .03 | -.03 | .04 | .04 | .05 |
| O2 Aesthetics | .03 | .00 | .02 | .11 | .02 | .00 |
| O3 Feelings | .07 | .05 | .08 | .12 | .06 | .05 |
| O4 Actions | -.01 | .03 | .01 | .12 | .10 | .04 |
| O5 Ideas | -.05 | .03 | -.03 | .08 | .19^*^ | .22^*^ |
| O6 Values | .02 | .08 | .06 | .05 | .19^*^ | .18^*^ |
| A1 Trust | .00 | -.02 | -.01 | .11 | .14^*^ | .06 |
| A2 Straightforwardness | .07 | -.04 | .04 | .08 | .03 | -.07 |
| A3 Altruism | .05 | -.05 | .01 | -.01 | .00 | .03 |
| A4 Compliance | -.09 | -.06 | -.10 | .02 | -.06 | -.11 |
| A5 Modesty | -.02 | -.04 | -.04 | -.07 | -.15^*^ | -.16^*^ |
| A6 Tender-mindedness | .04 | .02 | .04 | .13^*^ | .03 | .03 |
| C1 Competence | .16^*^ | .08 | .17^*^ | .22^*^ | .30^*^ | .28^*^ |
| C2 Order | .09 | .01 | .08 | .14^*^ | .08 | -.01 |
| C3 Dutifulness | .17^*^ | .01 | .14^*^ | .06 | .16^*^ | .08 |
| C4 Achievement-Striving | .20^*^ | -.01 | .16^*^ | .14^*^ | .18^*^ | .16^*^ |
| C5 Self-Discipline | .13 | .09 | .15^*^ | .23^*^ | .27^*^ | .18^*^ |
| C6 Deliberation | .16^*^ | .02 | .15^*^ | .14^*^ | .13^*^ | .11 |

LPA=light physical activity, MVPA=moderate-to-vigorous physical activity, MET=metabolic equivalent, *p<.05
