# Supplementary material for: Personality traits and physical functioning: a cross-sectional multimethod facet-level analysis
Source: Eur Rev Aging Phys Act. 2020 Nov 24;17:20. doi: 10.1186/s11556-020-00251-9 (PMC7685629; doi:10.1186/s11556-020-00251-9)
Supplement: Supplementary file 4 — Additional file 4: Table S4. Associations of personality traits and facets with accelerometer-assessed moderate-to-vigorous physical activity. Description: Results of the regression analyses for accelerometer-assessed moderate-to-vigorous physical activity. [file 11556_2020_251_MOESM4_ESM.docx]

Table S4. Associations of personality traits and facets with accelerometer-assessed moderate-to-vigorous physical activity.

|  | M1 | M2 |
| --- | --- | --- |
| Neuroticism | .08 | .12 |
| Extraversion | -.05 | -.06 |
| Openness | .10 | .08 |
| Agreeableness | .02 | -.02 |
| Conscientiousness | -.01 | -.06 |
| N1 Anxiety | .12 | .12 |
| N2 Angry Hostility | .10 | .10 |
| N3 Depression | .08 | .12 |
| N4 Self-Consciousness | .08 | .10 |
| N5 Impulsiveness | -.04 | .02 |
| N6 Vulnerability | .02 | .04 |
| E1 Warmth | -.12 | -.10 |
| E2 Gregariousness | -.07 | -.05 |
| E3 Assertiveness | -.03 | -.05 |
| E4 Activity | .03 | .02 |
| E5 Excitement Seeking | .04 | .03 |
| E6 Positive Emotions | -.06 | -.04 |
| O1 Fantasy | .05 | .05 |
| O2 Aesthetics | .08 | .08 |
| O3 Feelings | .13 | .13 |
| O4 Actions | .03 | -.01 |
| O5 Ideas | .02 | -.01 |
| O6 Values | .09 | .02 |
| A1 Trust | -.00 | -.03 |
| A2 Straightforwardness | -.02 | -.05 |
| A3 Altruism | -.06 | -.03 |
| A4 Compliance | -.03 | -.03 |
| A5 Modesty | .01 | .01 |
| A6 Tender-mindedness | .05 | .05 |
| C1 Competence | -.01 | -.05 |
| C2 Order | -.01 | -.04 |
| C3 Dutifulness | .00 | -.03 |
| C4 Achievement-Striving | -.04 | -.07 |
| C5 Self-Discipline | .03 | -.01 |
| C6 Deliberation | .00 | -.03 |

Every trait and facet analyzed in the separate linear regression model; standardized beta-coefficients represented. M1= model including sex and age as covariates. M2=model including sex, age, light physical activity, education, BMI, diseases and intervention group. *p<.05.
